# A Resourceful Work Environment Moderates the Relationship between Presenteeism and Health. A Study Using Repeated Measures in the Swedish Working Population

**DOI:** 10.3390/ijerph17134711

**Published:** 2020-06-30

**Authors:** Gunnar Bergström, Klas Gustafsson, Emmanuel Aboagye, Staffan Marklund, Gunnar Aronsson, Christina Björklund, Constanze Leineweber

**Affiliations:** 1 Department of Occupational Health Sciences and Psychology, Centre for Musculoskeletal Research, University of Gävle, 801 76 Gävle, Sweden; gunnar.bergstrom@hig.se (G.B.); emmanuel.aboagye@hig.se (E.A.); 2 Institute of Environmental Medicine, Unit of Intervention and Implementation Research for Worker Health, Karolinska Institutet, 171 77 Stockholm, Sweden; christina.bjorklund@ki.se; 3 Department of Clinical Neuroscience, Division of Insurance Medicine, Karolinska Institutet, 171 77 Stockholm, Sweden; klas.gustafsson@ki.se (K.G.); staffan.marklund@ki.se (S.M.); 4 Department of Psychology, Stockholm University, 106 91 Stockholm, Sweden; gunnar.aronsson@psychology.su.se; 5 Stress Research Institute, Stockholm University, 106 91 Stockholm, Sweden

**Keywords:** presenteeism, work environment, health protective, Sweden, job demands, job resources

## Abstract

The objective of this study was to investigate if the psychosocial work environment moderates the proposed negative impact of presenteeism on future general health. We expect that the negative impact of presenteeism on general health is weaker if the psychosocial work environment is resourceful, and more pronounced if the environment is stressful. Data were derived from the 2008–2018 biennial waves of the Swedish Longitudinal Occupational Survey of Health (SLOSH). The final analytic sample consisted of *n* = 15,779 individuals. We applied repeated measures regression analyses through generalized estimating equations (GEE). Results from the autoregressive GEE models showed statistically significant interaction terms between presenteeism and all four investigated moderators, i.e., job demands, job control, job support and job strain. The results indicate that the psychosocial work environment moderates the negative association between presenteeism and general health and illustrates a buffering effect of the psychosocial work environment. A possible explanation for these results may be that psychosocially resourceful work environments give room for adjustments in the work situation and facilitate recovery. The results also indicate that by investing the psychosocial work environment employers may be able to promote worker health as well as prevent reduced job performance due to presenteeism.

## 1. Introduction

Presenteeism, that is, the behaviour of going to work despite illness, has attracted increasing interest from occupational health researchers during the two last decades [1]. By definition, presenteeism is caused by ill health but research has shown that many other factors, both occupational and individual, are involved in the decision to go to work or stay home when sick. Examples of attendance pressure factors at work are time pressure, high job demands, role conflicts, job insecurity or having to catch up work if absent [1,2]. Individual factors related to presenteeism have been investigated to a lesser degree than occupational factors but characteristics such as performance-based self-esteem [3,4] and optimism [5] have been suggested to increase the tendency to go to work when ill.

A number of original studies and reviews have shown that presenteeism can increase the risk of future health problems such as mental and somatic symptoms as well as sick leave [6,7]. In a study by Conway et al. [8] presenteeism of more than eight days in the year previous to baseline was an independent predictor of depressive symptoms two years later. Likewise, Lu, Lin and Cooper [9] showed that presenteeism was related to poorer mental and physical health at a two-month follow-up. Moreover, Gustafsson and Marklund [10] showed that presenteeism predicted future physical complaints, poor health, reduced mental well-being and low work ability (after one year). Regarding sickness absence, it has been found that presenteeism is an independent predictor of long-term sickness absence over such long periods as 18 months [11,12]. In summary, studies indicate that the act of presenteeism increases the risk of future poor health and it seems that this finding is generalizable across occupational groups or lengths of follow up periods.

The phenomenon described in the former paragraph may be associated with non-adaptive or “negative” presenteeism, that is, when employees turn up sick at work, e.g., due to attendance pressures or organizational restrictions for sickness absence, even though they would have benefitted from recovering at home [13,14]. In fact, all twelve longitudinal studies that were included in the above cited systematic review on the impact of presenteeism on health and well-being departed from a health deleterious perspective on presenteeism [7]. Despite this, the question appears if presenteeism under certain circumstances could be beneficial for health [13,14,15,16]; for example, a mildly depressed person might gain from the social situation at work. It seems reasonable that the type of health condition as well as the work environment need to be considered for this purpose.

Both from a health perspective and an organizational perspective, employees with severe diseases that demand rest should not go to work and employees with acute communicable diseases should stay at home for the protection of their own health, their colleagues and the productivity of the organization. However, “going to work while ill” may be helpful for employees with less severe illnesses and/or manageable chronic illness, provided that the demands at work are reasonable and that job resources are available. There are several indicators which would support such a stance; for example, evidence-based recommendations for common back pain emphasize the importance of maintaining normal activities, including gainful work, to lower the risk of disability [17]. For Common Mental Disorders (CMDs) the importance of returning to work despite remaining psychological symptoms is also considered as crucial to avoid negative long-term effects such as prolonged sick leave, unemployment, social isolation and financial problems [18,19,20]. However, the availability of resources and support at work can be expected to be of vital importance when an employee who does not feel well goes to work [18,19] 

To the best of the authors’ knowledge there exists no published comprehensive longitudinal study of the potential moderating impact of psychosocial job resources on the relation between presenteeism and health. However, in a cross-sectional study from Norway, Lau et al. [21] found that employees going to work despite common mental disorders (CMDs) described a better psychosocial work environment, less symptoms and better well-being and functioning as compared to employees on full-time sick leave. Furthermore, based on an assessment by their therapists, those staying at work were also described as functioning better than a group of employees that were partially on sick leave due to CMDs. Still, conclusions about causal relationships between the variables are hampered by the study’s cross-sectional design.

In a conceptual paper departing from several psychological and/or psychosocial theories [22,23,24], Karanika-Murray and Biron [14] focus on the adaptive potential of presenteeism. According to the cited paper four “types” of presenteeism can be delineated, two potentially adaptive forms of presenteeism and two potentially non-adaptive forms. A functional presenteeism is at hand when the health condition allows working despite symptoms and where job resources are available. Such job resources can be latitude to adjust work tasks, job support, opportunities to delegate work, etc., that enable the employee to work within the boundaries of their health problem. In such a situation going to work while ill could be beneficial for the employee’s health and wellbeing simultaneously as the presentee attends to existing job duties. Another type of adaptive presenteeism is described as “therapeutic” and in this case the employee does not primarily contribute to any substantial part of the “production” at a workplace but presenteeism can be beneficial to the employee due to the social aspects of the work and because of the activity and variation going to work implies. Even here job resources are important by enabling being at work despite the employees’ work performance being far below the normal level. There are also two types of presenteeism assumed to be non-adaptive [14], of which one is a dysfunctional type where the health condition demands rest and recuperation and where job performance is low. Finally, an “overachieving” type of presenteeism is described where the work performance can be high but in the long run undermine the health of the employee due to continuous arousal and lack of recovery. Additionally, these four types of presenteeism are described as dynamic and changing, and presentees can pass from one type to another depending on their health condition and/or the availability of job resources [14].

According to Karanika-Murray and Biron [14], when an employee works while ill this further accentuates the importance of job resources in the work environment to be able to meet the prevailing performance demands. This is also supported by empirical findings related to the job demands-resources (JD-R) theory where job resources have been found to buffer the impact of job demands on strain [22,25]. Consequently, the availability of job resources makes an adaptive presenteeism more probable. In the present study, we focus on two potentially resourceful work environments (high control and high job (social) support at work), one job situation with low job demands, as well as a job situation characterized by pressure and stress. The resourceful work environments are expected to offer more opportunities for work adjustments and recovery, initialized by either the employee or the employer, and may therefore contribute to a buffering effect on the expected negative health effect of presenteeism. For instance, employees with high control may to a higher degree be able to adjust their job situation (e.g., work pace) according to their needs, and employees with higher job support from colleagues or their supervisors will likely be more able to manage their job situation, e.g., by social support or by support with job tasks [14]. 

Employees with low work demands can be expected to have more time to do the job and more room for taking breaks and to recover even in situations where job resources are lacking. This is also in line with the JD-R theory, which proposes that job resources are especially important or salient when demands are high [22]. Finally, the stressful and less resourceful work environment used in this study was high job strain (a combination of high demands and low control) according to the demand–control model [26,27]. High strain jobs are more likely to be distinguished by pressure of time, demands to work fast and hard and a lack of the job resource decision authority and skill discretion. This may make this stressful job situation especially challenging for employees already suffering from poor health and thus more likely to be associated with an amplified negative health effect of presenteeism [14,22]. 

The aim of the study was to investigate if the psychosocial work environment measured in terms of job demand, control and support moderates the proposed negative impact of presenteeism on future general health. Specifically, we expect that the negative impact of presenteeism on general health is weaker if the psychosocial work environment is resourceful, and more pronounced if the environment is stressful. It may be added that this study is not an attempt to test the propositions by Karanika-Murray and Biron [14] specifically since this would also require information about the health condition of the presentee. However, we have been inspired by their [14] emphasis on the importance of a resourceful work environment and the flexibility this may offer employees going to work while ill.

## 2. Materials and Methods 

The study was based on data derived from the Swedish Longitudinal Occupational Survey of Health (SLOSH) study. SLOSH started in 2006 with a first follow up of participants of the Swedish Work Environment Survey (SWES) 2003. Further SWES cohorts have been added and today SLOSH comprises of all SWES participants 2003–2011 (*n* = 40,877). Since the start of SLOSH, a questionnaire in two versions has been sent out every second year, one for those in paid work and one for those who have temporarily or permanently left paid work (e.g., are on parental leave or retired). Detailed information on the study design, etc. is found elsewhere [28].

The current study is based on participants who answered the questionnaire for those in work at least at two subsequent times between 2008 (the first year presenteeism was measured; *n* = 15,783) and 2018, and who had valid information on sickness presenteeism or general health in at least one wave (*n* = 15,779). In the study sample, most participants (37%) had answered at three waves, about one quarter of all participants answered to only two waves and between 10% and 14% answered between four to six waves.

### 2.1. Measures

**Figure 1:**
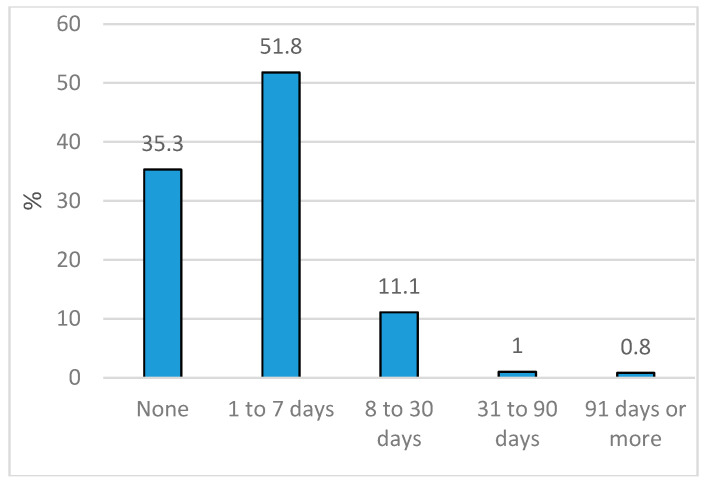
Distribution of presenteeism (days of going to work despite one should have reported in sick) in the study sample (SLOSH) 2008 (*n* = 7661).

The exposure variable sickness presenteeism was measured by a single question asking “For roughly how many days have you gone to work knowing that, owing to your condition, you ought to have reported in sick in the past 12 months?” Response options were: None, 1–7 days, 8–30 days, 31–90 days and 91 days or more. Before any further analyses, presenteeism was dichotomized into no vs. at least 1–7 days or more of sickness presenteeism during the past 12 months [29]. A distribution of response alternatives is shown in Figure 1.

The outcome was self-rated general health, a widely used measure of perceived current health status, and measured by the standard single-item question “How would you rate your general state of health?” [30]. The validity and reliability of this item has been shown in various studies and the item is considered a reliable and valid global health measure [31]. Respondents answered on a five-point scale ranging from “Very poor” to “Very good” with higher values indicating better general health. 

The moderator variables job demands, job control and job (social) support were measured by the Swedish Demand–Control-Support Questionnaire (DCSQ), which is based on Karasek’s Demand–Control-Support Model [27,32]. Job demands are measured by five items including both qualitative and quantitative aspects of work (Cronbach’s alpha: 0.72 to 0.74). The scale was reversed so that higher values indicate lower demands. The five questions measuring job control cover two areas: Skill discretion and decision authority (Cronbach’s alpha 0.66 to 0.67). Six questions on job support regard the atmosphere at work as well as support from colleagues and supervisors (Cronbach’s alpha: 0.82 to 0.87). All questions have a four-grade response scale from “never” to “always”. For analyses, mean scores of the items were calculated and the scores grand-mean centred. Higher mean values indicate more job control and job support, and lower job demands. All these moderators were used as continuous variables.

To construct a measure for job strain, psychological demands and job control were split by the median, the so called quadrant approach [33], and combined into four dimensions of jobs, that is: “Active jobs” (high demands and high control), “passive jobs” (low demands and low control), “low strain jobs” (low demands and high control) and “high strain jobs” (high demands and low control). For analyses, these were further dichotomized into “high strain” vs. all other types.

Age, sex, education and having a leading position were deemed to be potential confounding factors, as all have a strong influence on general health [34,35]. All confounders, except those with a leading position, were derived from linkage to registry data. Age is age at first observation. Sex is dichotomous (0 = men, 1 = female). Information on education was upper secondary education or lower vs. higher education. The item “Do you have a managerial position with personnel responsibilities?” was used to measure having a leading position. Response options in 2008 and 2010 were “yes” vs. “no”. From 2012, more response options were provided on this item and those who answered “Yes, I am a leader but not boss”, “Yes, I am boss without personnel responsibility” and “Yes, I am boss with personnel responsibility” were considered having a leading position.

### 2.2. Statistical Analyses Approach

Data were analysed using generalized estimating equations (GEE), a method for longitudinal data which allows for simultaneous analyses of variables at different time points [36]. GEE is, like mixed models, “highly suitable for the analysis of longitudinal data” [37] (p. 81). It adjusts for the dependency of the observations within one subject by assuming a certain working correlation structure and produces very similar results to mixed models. Identical-linked regression models with a normal distribution were specified to calculate regression coefficients. After inspection of the correlations of the dependent variable, analyses were performed using an exchangeable working correlation structure. As the standard GEE pool together between participants’ (cross-sectional) and within participants’ (longitudinal) relationships, we also applied autoregressive GEE to remove the between-subjects part of the relationship between sickness presenteeism and general health [33]. In autoregressive GEE the value of the outcome variable (here, general health) at a specific time point is predicted additionally by the outcome variable measured on time-point earlier, i.e., yt-1 (autoregression) [33]. GEE analyses were carried out in Proc Genmod in the statistical package SAS (version 9.4, SAS Institute Inc., Cary, NC, USA). This procedure can estimate the working correlation from data containing missing values (either due to dropout or intermittent missing values) by using the all available pairs method, in which all nonmissing pairs of data are used in the moment estimators of the working correlation parameters defined previously.

We analysed the relationship between sickness presenteeism and general health in several steps. First, sickness presenteeism and psychosocial work environment were analysed separately in their relation to general health (Model 0). Secondly, we added a multiplicative interaction term between sickness presenteeism and possible moderators, thus testing if sickness presenteeism is less harmful under more favourable working conditions (Model 1). In a last model (Model 2), we adjusted for age, sex, education and leading position. All variables except age and sex were used as time-varying confounders.

## 3. Results

Descriptive characteristics for all waves are presented in Table 1. Age at baseline, that is 2008, was on average 47.7 (SD = 10.1) years. Generally, more women than men participated in SLOSH. About half of the study population had post-secondary education and about one third reported having a leading position. Between 54% and 65% reported at least one day of sickness presenteeism during the past 12 months and between 18% and 21% reported intermediate or poor general health. As seen in Table 1 the proportion of those reporting intermediate or poor general health increased over time. All potential confounders, except sex, were independently associated with general health (see online supplement Table S1). Attrition analyses showed that a larger proportion of subjects who answered at all waves (*n* = 2153) as compared to those who did not answer at all waves (*n* = 13,626) were female (58.9% vs. 56.5%, *p* < 0.05) and reported at least one day of presenteeism during the past 12 months (64.2% vs. 59.0%, *p* < 0.001). They were also younger (45.7 years vs. 48.9 years) and reported slightly lower levels of job control (3.10 vs. 3.14, *p* < 0.01). No differences in education, leading position, self-rated health, job demand, job strain or job support were found.

Results of the generalized estimating equations (GEE) for standard GEEs and autoregressive GEEs are presented in Table 2 and Table 3. The autoregressive models provided similar results as the standard GEEs. In the autoregressive analyses, sickness presenteeism as well as job demands, job control, job support (Table 2, Model 0) and job strain (Table 3, Model 0) showed statistically significant associations with future general health in the crude models. Focusing on the “within subjects” interpretations of our results, sickness presenteeism showed a negative association with general health, indicating that the association between presenteeism and future general health can be explained by within-effects, that is, increase in presenteeism at a personal level relates to a decrease in general health in that person. For example, in the uncontrolled (autoregressive) model, we find a β-value of −0.17 for sickness presenteeism, indicating that a one-unit increase in sickness presence is associated with a 0.17 units decrease in general health. Introducing an interaction term revealed the main effects and statistically significant interaction terms between sickness presenteeism and all four indicators of the work environment (unadjusted autoregressive models). The interactions terms reached statistical significance for job demands (Table 2, Model 1a: β = 0.07, *p* < 0.0001), job control (Table 2, Model 2a: β = 0.08; *p* < 0.0001), job support (Table 2, Model 3a β = 0.08, *p* < 0.0001) and job strain (Table 3, Model 1: β = −0.08; *p* < 0.0001). Thus, our results indicate that the negative relationship between sickness presenteeism and general health is less pronounced with increasing levels of job control and support and decreasing levels of job demands. In other words, for a person who experiences sickness presenteeism under low job demands, the negative effect of presenteeism on general health is on an average reduced by 0.07 points. Controlling for age, sex, education and having a leading position did not change the results notably (see Table 2, Model 1b–3b and Table 3, Model 2). Sensitivity analyses treating presenteeism as a continuous variable provided very similar results (see Online Supplement Table S2).

In additional analyses, we investigated how health develops over time and if this development differs according to presenteeism. The analyses revealed that time has a major effect on health (β = −0.026, 95% CI: −0.029 to −0.022), indicating that health generally declined over time. However, the development was the same independent of sickness presenteeism; that is, there were no interaction effects between sickness presenteeism and time.

Finally, trying to further understand the contribution of the different moderators, we run a model where we included all interaction terms simultaneously. In this model, all the main effects remained statistically significant as did all moderators except job strain (see Table 4). However, the moderating effect of job control was considerably attenuated in this model and reached only marginally statistical significance (*p* = 0.05 in both the unadjusted and the adjusted models).

## 4. Discussion

### 4.1. Key Results

The aim of this study was to investigate the potential moderating effects of the psychosocial work environment on the proposed negative impact of presenteeism on general health. Specifically, we were interested in whether this proposed negative effect is less pronounced when the work environment contains more psychosocial job resources and thus can buffer the negative effects of working while ill. To test this idea, we use a large dataset approximately representative for the working population in Sweden and employed a longitudinal analysis technique, which allowed an efficient adjustment for correlated data. The results showed (1) that going to work while ill was consistently associated with a decline in future general health and (2) that employees with presenteeism who reported more available job resources in terms of high job control and support or employees reporting lower job demands showed better future general health than a reference group with the same amount of presenteeism but with less job control/support or higher demands at work, respectively. Furthermore, employees engaged in presenteeism in a stressful work environment with high job demands and low job control (job strain) described worse general health compared to employees with the same amount of presenteeism who were working under less stressful circumstances. Furthermore, when all interactions and work environment variables were included in one single model all moderators except job strain independently associated with a buffering effect of the negative health impact of presenteeism. In other words, we found that a resourceful psychosocial work environment and a work environment with low work demands was associated with better future general health among employees who reported presenteeism.

### 4.2. Theoretical and Practical Implications

According to Karanika-Murray and Biron [14] employees working while ill in a job situation with more available job resources (in this study, high control and social support) can be expected to have more room for adjustments such as varying the work pace, doing the job in a different way, taking breaks or choosing different work tasks. Furthermore, working in a situation of high job support may ease the situation, for example, as colleagues take on certain work tasks. It may also be that the sense of control and support in itself may make the situation less arduous or distressing. These different aspects of job resources could probably be supportive to the employee’s health. Similarly, employees with lower demands can be expected to have more opportunities for recovery at work, and a less pressing work situation may make the need for job resources less prominent [22]. On the contrary, for sickness to be present in a stressful work environment characterized by high demands and low control (i.e., job strain) is a well-known risk factor for ill health [26] and may be certainly counterproductive for recovery among employees engaged in presenteeism as they already have a health problem to cope with. This stressful work situation does also correspond to the health impairment process described by the JD-R theory where employee health is eroded by the combination of high demands and insufficient resources [22]. It should also be noted that in the full model where all interactions and variables were included only job support and job demands showed to be distinct independent moderators of the presenteeism–health association. This may indicate that high job support and lower work demands are certainly important as buffers of negative health effects from presenteeism.

The results in this study bring some support to the proposed assumptions concerning the potential of job resources to protect against negative health effects of presenteeism [14]. Consequently, in a state of poor health, job resources as well as reasonable demands become increasingly salient and valuable for preventing further poor health for the presentee. However, if employees, despite health problems, go to work whilst their job situation allows for work adjustments, this may prevent some health deterioration associated with presenteeism, but nevertheless a general health decline was shown among presentees, though to a lesser degree among those with more job resources. It should also be noted that being a presentee in a stressful work environment further reinforced the negative health effect of presenteeism. It could be that the health conditions behind the presenteeism may explain some of this result (the health reasons directly associated with presenteeism were not measured).

The item used to measure presenteeism in this study indicates that the reported presenteeism is, at least to some degree, involuntary (the item includes “ought to have reported in sick”) [29]. It is not clear how this might have influenced the results. If an employee engages in presenteeism involuntarily it may indicate that their health condition is not suitable for working and/or that the work environment does not offer adequate resources or adjustments for adaptive presenteeism [16]. However, it may also be that even if employees believe that it is not good to go to work because of their health problem, presenteeism nevertheless might have helped them to regain health to some degree if the psychosocial work environment contained supporting job resources. There could be “grey zones” where, for instance, the work situation contains realistic and feasible opportunities for adjustments and has the potential to be health promoting for some presentees, but, nevertheless, the employee might be hesitant about going to work.

### 4.3. Strengths

We used a large approximately representative population of the Swedish working population and a longitudinal design to investigate moderating effects of the work environment on the presenteeism–general health relationship. While the analytical technique we used provides more robust results than the traditional regression analysis, it does not allow conclusions about causation or the direction of relationships. The outcome used in this study, general health, is closely related to presenteeism as presenteeism requires by definition some amount of poor health. This means that the frequency of presenteeism may be lower at the follow-ups for those with improved health (or less deteriorated health). We addressed this issue to some degree by adjusting for previous general health in the analyses. The autoregressive analyses indicate that changes in the one factor (e.g., presenteeism) relate to changes in the other factor (e.g., general health). The effect cannot be explained solely by differences between persons since this method takes correlations within individuals over time into account and allows for an efficient adjustment of correlated data.

### 4.4. Limitations

Problems may arise from missing data and drop out as it is possible that the distribution of the observed data is not the same as the distribution of the complete data. It has been suggested that the GEE models may yield biased estimates unless the drop out is MCAR (missing completely at random). However, it seems that outcomes are generally comparable between GEE and techniques that assume data are missing at random (MAR), as, for example, mixed models, or even missing not at random (MNAR) [37]. Moreover, only a few differences between subjects without any missing data over the longitudinal period and subjects with missing data at one or more of the repeated measurements indicated only a few statistically significant differences (that most probably can be explained by the large sample size). Another limitation might be found in the relatively low reliability of the job control measure. However, while a previous publication suggests the exclusion of a certain variable [38], we decided to stick to the common measure to allow for better comparability between different studies. Moreover, subjects could have changed job between the study waves and thus improve (or worsen) their job situation. However, we would expect that such changes would have a similar influence on general health and presenteeism, such as not changing the nature of the association. In this study we have not considered the potential health problems that lie behind the presenteeism. This probably meant that our estimates were diluted by mixing employees where presenteeism could be harmful with employees where presenteeism may promote health.

Admittedly, a more detailed screening of both the work situation and individual factors for employees reasonably could add a lot to our understanding of the consequences of presenteeism. While the comprehensiveness of our study sample allows generalisability, it may be worthwhile to study specific sectors of employment or occupations, as demands, control and job support can take quite different forms in different work environments. Moreover, our findings might not be generalizable to different working cultures, as, e.g., the working culture of Eastern countries. A critical reflection should also be added concerning the definition of high versus low, control, support and demands in this study. Since we used split by the median to form these groups the terms “high” and “low” are relative, and it may, for instance, be that many employees in the low demand group do not perceive the demands at work as low, but as reasonable.

### 4.5. Future Research

One goal with future research would be to develop an empirically based and theoretically sound model that could inform research and occupational health practice about under which conditions presenteeism may be adaptive and when presenteeism is non-adaptive. To do this, theoretical frameworks such as the one presented by Karanika-Murray and Biron [14] should be empirically evaluated and measurement tools that are helpful to define different adaptive or non-adaptive types of presenteeism need to be developed. To understand, and predict, the future consequences of presenteeism it may also be useful to assess employees’ reasons for being sickness present [2], along with their expectations concerning the consequences of engaging in presenteeism or the alternative to being sickness absent. Earlier research from other areas has shown that individuals’ expectations/beliefs about the future health status or sickness absence can be more accurate than expert predictions [39].

## 5. Conclusions

The results of the present study show that a resourceful psychosocial work environment had a buffering impact on the health impairing effect of presenteeism. Consequently, it seems reasonable that the resourceful work environments evaluated here lend room for, e.g., work adjustments and recovery at work and that this prevents some of the negative effects of presenteeism. This points to an opportunity for employers since by investing in the psychosocial work environment they may be able to promote worker health as well as prevent reduced job performance due to presenteeism among the employees. Finally, there is a need to develop more sophisticated measurements of presenteeism that enable evaluation of the potentially functional and dysfunctional sides of presenteeism.

## Figures and Tables

**Table 1:** Characteristics of the study participants at wave 2 (2008)–wave 7 (2018).

Variables	Wave 2 *n* = 7661	Wave 3 *n* = 8246	Wave 4 *n* = 7227	Wave 5 *n* = 13,103	Wave 6 *n* = 13,629	Wave 7 *n* = 11,933
**Sex, *n* (%)**						
Male	3298 (43.0)	3557 (43.1)	3098 (42.9)	5512 (42.1)	5795 (42.5)	4979 (41.7)
Female	4363 (57.0)	4689 (56.9)	4129 (57.1)	7591 (57.9)	7834 (57.5)	6954 (58.3)
**Education, *n* (%)**						
≤post-secondary education	4087 (53.4)	4295 (52.1)	3713 (51.4)	6281 (48.0)	6421 (47.1)	5496 (46.0)
>post-secondary educations	3571 (46.6)	3949 (47.9)	3513 (48.6)	6817 (52.0)	7202 (52.9)	6428 (54.0)
**Leading position, *n* (%)**						
Yes	2468 (34.3)	2680 (34.4)	1951 (30.9)	3795 (33.4)	3890 (33.1)	2961 (32.6)
**Sickness presenteeism, *n* (%)**						
no day during the past 12 months	2667 (35.3)	2974 (37.9)	2601 (37.8)	5125 (39.8)	5344 (40.1)	5213 (45.5)
at least 1 day during the past 12 months	4883 (64.7)	4876 (62.1)	4280 (62.2)	7760 (60.2)	7973 (59.9)	6240 (54.5)
**General health; *n* (%)**						
Very good or good	6244 (82.1)	6522 (80.2)	5751 (80.0)	10,482 (80.7)	10,767 (79.7)	9338 (78.7)
Intermediate or poor	1364 (17.9)	1612 (19.8)	1440 (20.0)	2511 (19.3)	2746 (20.3)	2531 (21.3)

**Table 2:** Results of standard and autoregressive generalized estimating equations (GEE) analyses of the association between sickness presenteeism (1 = sickness presenteeism of >= 1 day during the past 12 months) and general health, presented as beta estimates (β) with 95% CIs, taking job demands, job control and job support as possible moderating job characteristics into account.

Results for standard GEE	Model 0		Model 1a		Model 1b		Model 2a		Model 2b		Model 3a		Model 3b	
Variables	β	95% CI	β	95% CI	β	95% CI	β	95% CI	β	95% CI	β	95% CI	β	95% CI
Sickness presenteeism	−0.18	−0.19; −0.17	−0.17	−0.18; −0.15	−0.21	−0.22; −0.19	−0.18	−0.19; −0.16	−0.22	−0.24; −0.21	−0.21	−0.22; −0.19	−0.21	−0.23; −0.20
Low job demands	0.18	0.16; 0.19	0.12	0.10; 0.13	0.12	0.10; 0.14								
High job control	0.12	0.10; 0.13					0.07	0.05; 0.09	0.11	0.08; 0.13				
High job support	0.21	0.20; 0.22									0.15	0.14; 0.17	0.16	0.14; 0.18
Presenteeism*demands			0.08	0.06; 0.10	0.09	0.06; 0.11								
Presenteeism*control							0.08	0.05; 0.10	0.05	0.03; 0.08				
Presenteeism*support											0.07	0.04; 0.09	0.06	0.04; 0.09
**Results for** **autoregressive GEE**	**β**	**95% CI**	**β**	**95% CI**	**β**	**95% CI**	**β**	**95% CI**	**β**	**95% CI**	**β**	**95% CI**	**β**	**95% CI**
Sickness presenteeism	−0.17	−0.19; −0.16	−0.16	−0.17; −0.14	−0.19	−0.21; −0.18	−0.17	−0.18; −0.16	−0.21	−0.22; −0.20	−0.19	−0.20; −0.18	−0.20	−0.21; −0.18
Low job demands	0.13	0.12; 0.14	0.07	0.05; 0.08	0.07	0.05; 0.09								
High job control	0.09						0.03	0.01; 0.05	0.05	0.03; 0.08				
High job support	0.21	0.20; 0.22									0.11	0.09; 0.14	0.11	0.09; 0.13
General health at t-1			0.56	0.55; 0.58	0.56	0.55; 0.57	0.58	0.57; 0.59	0.57	0.56; 0.58	0.55	0.54; 0.56	0.55	0.54; 0.56
Presenteeism*demands			0.07	0.04; 0.09	0.08	0.05; 0.10								
Presenteeism*control							0.08	0.05; 0.11	0.06	0.03; 0.09				
Presenteeism*support											0.08	0.05; 0.10	0.08	0.05; 0.10

Model 0: Crude model; Model a: Added interaction term; Model b: Adjusted for age, sex, education, and leading position.

**Table 3:** Results of standard and autoregressive generalized estimating equation (GEE) analyses of the association between sickness presenteeism (1 = sickness presenteeism of >= 1 day during the past 12 months) and general health, presented as beta estimates (β) with 95% CIs, taking job strain as a possible moderating job characteristic into account.

Results for standard GEE	Model 0		Model 1		Model 2	
**Variables**	**β**	**95% CI**	**β**	**95% CI**	**β**	**95% CI**
Sickness presenteeism	−0.18	−0.19; −0.17	−0.16	−0.17; −0.14	−0.20	−0.22; −0.19
Strain	−0.16	−0.18; −0.15	−0.10	−0.12; −0.08	−0.11	−0.13; −0.08
Presenteeism*strain			−0.07	−0.09; −0.04	−0.07	0.10; −0.04
**Results for autoregressive GEE**	**β**	**95% CI**	**β**	**95% CI**	**β**	**95% CI**
Sickness presenteeism	−0.17	−0.19; −0.16	−0.14	−0.16; −0.13	−0.18	−0.20; −0.17
Strain	−0.16	−0.17; −0.14	−0.08	−0.10; −0.06	−0.08	−0.11; −0.06
General health at t-1			0.57	0.56; 0.58	0.57	0.56; 0.58
Presenteeism*strain			−0.08	−0.11; −0.04	−0.08	−0.11; −0.05

Model 0: Crude model; Model 1: Added interaction term; Model 2: Adjusted for age, sex, education and leading position.

**Table 4:** Results of the autoregressive adjusted generalized estimating equation (GEE) analyses of the association between sickness presenteeism (1 = sickness presenteeism of >= 1 day during the past 12 months) and general health, presented as beta estimates (β) with 95% CIs, taking all possible moderating job characteristics into account.

Results for standard GEE	Model 1		Model 2	
Variables	β	95% CI	β	95% CI
Sickness presenteeism	−0.18	−0.19; −0.16	−0.18	−0.20; −0.16
Low job demands	0.03	0.05; 0.01	0.04	0.06; 0.02
High job control	0.04	0.02; 0.06	0.03	0.01 0.05
High job support	0.10	0.08; 0.12	0.10	0.07; 0.12
Job strain	−0.03	−0.06; 0.00	−0.02	−0.05; 0.01
General health at t-1	0.54	0.53; 0.55	0.54	0.52; 0.55
Presenteeism*demands	−0.05	−0.08; −0.02	−0.05	−0.8; −0.02
Presenteeism*control	0.03	0.00; 0.06	0.03	0.00; 0.06
Presenteeism*support	0.05	0.02; 0.08	0.05	0.02; 0.08
Presenteeism*strain	−0.02	−0.06; 0.02	−0.03	−0.07; 0.01

Model 1: Mutually adjusted for all interactions simultaneously; Model 2: Additionally adjusted for age, sex, education and leading position.

## Supplementary Materials

The following are available online at https://www.mdpi.com/1660-4601/17/13/4711/s1, Table S1: Correlations among study variables, Table S2: Results of standard and autoregressive generalized estimating equations (GEE) analyses of the association between sickness presenteeism (continuous) and general health, presented as beta estimates (β) with 95% CIs, taking job demands, job control, and job support as possible moderating job characteristics into account
