# Supplementary material for: A Resourceful Work Environment Moderates the Relationship between Presenteeism and Health. A Study Using Repeated Measures in the Swedish Working Population
Source: Int J Environ Res Public Health. 2020 Jun 30;17(13):4711. doi: 10.3390/ijerph17134711 (PMC7370150; doi:10.3390/ijerph17134711)
Supplement: Supplementary file 1 [file ijerph-17-04711-s001.zip › New folder/ijerph-832514-SUPP 2.docx]

|  | **Model 0** | | **Model 1a** | | **Model 1b** | | **Model 2a** | | **Model 2b** | | **Model 3a** | | **Model 3b** | | **Model 4a** | | **Model 4b** | |
| --- | --- | --- | --- | --- | --- | --- | --- | --- | --- | --- | --- | --- | --- | --- | --- | --- | --- | --- |
| **Results for**  **standard GEE** | **β** | **95% CI** | **β** | **95% CI** | **β** | **95% CI** | **β** | **95% CI** | **β** | **95% CI** | **β** | **95% CI** | **β** | **95% CI** | **β** | **95% CI** | **β** | **95% CI** |
| Sickness presenteeism | -0.21 | −0.22; −0.20 | -0.19 | −0.21; −0.19 | -0.23 | −0.24; −0.22 | -0.21 | -0.22; -0.20 | -0.25 | −0.26; −0.24 | -0.23 | −0.24; −0.22 | -0.24 | −0.25; −0.23 | −0.19 | −0.20; −0.18 | −0.23 | −0.24; −0.22 |
| Low job demands | 0.18 | 0.16; 0.19 | 0.14 | 0.15; 0.12 | 0.14 | 0.16; 0.13 |  |  |  |  |  |  |  |  |  |  |  |  |
| High job control | 0.12 | 0.10; 0.13 |  |  |  |  | 0.10 | 0.08; 0.11 | 0.13 | 0.11; 0.15 |  |  |  |  |  |  |  |  |
| High job support | 0.21 | 0.20; 0.22 |  |  |  |  |  |  |  |  | 0.17 | 0.16; 0.19 | 0.17 | 0.16; 0.19 |  |  |  |  |
| High job strain |  |  |  |  |  |  |  |  |  |  |  |  |  |  | --0.12 | −0.14; −0.11 | --0.12 | −0.14; −0.11 |
| Presenteeism*demands |  |  | 0.06 | 0.07; 0.04 | 0.06 | 0.08; 0.04 |  |  |  |  |  |  |  |  |  |  |  |  |
| Presenteeism*control |  |  |  |  |  |  | 0.04 | 0.02; 0.03 | 0.02 | 0.00; 0.04 |  |  |  |  |  |  |  |  |
| Presenteeism*support |  |  |  |  |  |  |  |  |  |  | 0.04 | 0.03; 0.06 | 0.04 | 0.03; 0.06 |  |  |  |  |
| Presenteeism*strain |  |  |  |  |  |  |  |  |  |  |  |  |  |  | −0.05 | −0.07; −0.03 | −0.05 | −0.07; −0.03 |
| **Results for**  **autoregressive GEE** | **β** | **95% CI** | **β** | **95% CI** | **β** | **95% CI** | **β** | **95% CI** | **β** | **95% CI** | **β** | **95% CI** | **β** | **95% CI** | **β** | **95% CI** | **β** | **95% CI** |
| Sickness presenteeism | -0.19 | −0.20; −0.18 | -0.18 | −0.19; −0.17 | -0.20 | −0.2123; −0.1917 | -0.19 | -0.20; -0.18 | -0.22 | −0.23; −0.21 | -0.20 | −0.21; −0.19 | -0.20 | −0.21; −0.19 | −0.17 | −0.18; −0.16 | −0.20 | −0.21; −0.18 |
| Low job demands |  |  | 0.08 | 0.10; 0.07 | 0.09 | 0.11; −0.08 |  |  |  |  |  |  |  |  |  |  |  |  |
| High job control |  |  |  |  |  |  | 0.07 | 0.06; 0.09 | 0.09 | 0.07; 0.11 |  |  |  |  |  |  |  |  |
| High job support |  |  |  |  |  |  |  |  |  |  | 0.14 | 0.13; 0.15 | 0.14 | 0.12; 0.15 |  |  |  |  |
| High job strain |  |  |  |  |  |  |  |  |  |  |  |  |  |  | −0.10 | −0.12; −0.09 | −0.11 | −0.12; −0.09 |
| General health at t-1 | 0.54 | 0.53; 0.55 | 0.52 | 0.51; 0.53 | 0.51 | 0.50; 0.52 | 0.53 | 0.52; 0.54 | 0.52 | 0.51; 0.53 | 0.50 | 0.49; 0.52 | 0.50 | 0.49; 0.52 | 0.53 | 0.52; 0.54 | 0.51 | 0.50; 0.52 |
| Presenteeism*demands |  |  | 0.06 | 0.07; 0.04 | 0.06 | 0.07; 0.04 |  |  |  |  |  |  |  |  |  |  |  |  |
| Presenteeism*control |  |  |  |  |  |  | 0.04 | 0.02; 0.06 | 0.03 | 0.03; 0.05 |  |  |  |  |  |  |  |  |
| Presenteeism*support |  |  |  |  |  |  |  |  |  |  | 0.04 | 0.03; 0.06 | 0.04 | 0.03; 0.06 |  |  |  |  |
| Presenteeism*job strain |  |  |  |  |  |  |  |  |  |  |  |  |  |  | −0.05 | −0.07; −0.03 | −0.05 | −0.07; −0.02 |

**Table S2.** Results of standard and autoregressive generalized estimating equations (GEE) analyses of the association between sickness presenteeism (continuous) and general health, presented as beta estimates (β) with 95% CIs, taking job demands, job control, and job support as possible moderating job characteristics into account.

0: crude model; Model a: added interaction term; Model b: added age, sex, education, and leading position.
